# Genomic data mining reveals a rich repertoire of transport proteins in *Streptomyces*

**DOI:** 10.1186/s12864-016-2899-4

**Published:** 2016-08-22

**Authors:** Zhan Zhou, Ning Sun, Shanshan Wu, Yong-Quan Li, Yufeng Wang

**Affiliations:** 1 College of Pharmaceutical Sciences, Zhejiang University, Hangzhou, 310058 People’s Republic of China; 2 Zhejiang Provincial Key Laboratory of Microbial Biochemistry and Metabolism Engineering, Zhejiang University, Hangzhou, 310058 People’s Republic of China; 3 Department of Biology and South Texas Center for Emerging Infectious Diseases, University of Texas at San Antonio, San Antonio, TX 78249 USA

**Keywords:** *Streptomyces*, Transport proteins, Comparative genomics, Drug efflux, Protein translocation

## Abstract

**Background:**

Streptomycetes are soil-dwelling Gram-positive bacteria that are best known as the major producers of antibiotics used in the pharmaceutical industry. The evolution of exceptionally powerful transporter systems in streptomycetes has enabled their adaptation to the complex soil environment.

**Results:**

Our comparative genomic analyses revealed that each of the eleven *Streptomyces* species examined possesses a rich repertoire of from 761-1258 transport proteins, accounting for 10.2 to 13.7 % of each respective proteome. These transporters can be divided into seven functional classes and 171 transporter families. Among them, the ATP-binding Cassette (ABC) superfamily and the Major Facilitator Superfamily (MFS) represent more than 40 % of all the transport proteins in *Streptomyces*. They play important roles in both nutrient uptake and substrate secretion, especially in the efflux of drugs and toxicants. The evolutionary flexibility across eleven *Streptomyces* species is seen in the lineage-specific distribution of transport proteins in two major protein translocation pathways: the general secretory (Sec) pathway and the twin-arginine translocation (Tat) pathway.

**Conclusions:**

Our results present a catalog of transport systems in eleven *Streptomyces* species. These expansive transport systems are important mediators of the complex processes including nutrient uptake, concentration balance of elements, efflux of drugs and toxins, and the timely and orderly secretion of proteins. A better understanding of transport systems will allow enhanced optimization of production processes for both pharmaceutical and industrial applications of *Streptomyces*, which are widely used in antibiotic production and heterologous expression of recombinant proteins.

**Electronic supplementary material:**

The online version of this article (doi:10.1186/s12864-016-2899-4) contains supplementary material, which is available to authorized users.

## Background

*Streptomyces* is a group of soil-dwelling Gram-positive bacteria, which are well known for their ability to produce a broad array of secondary metabolites including antibiotics, antifungals, antiparasitic drugs, anticancer agents, immunosuppressants, and herbicides [1, 2]. They are also ideal systems in biotechnology for heterologous expression of recombinant proteins with simple downstream processing and high yields [3, 4]. In order to survive in the complex soil environment, streptomycetes have evolved exceptionally powerful transport systems [5, 6]. For example, in *Streptomyces coelicolor*, there are more than 600 predicted transport proteins with a large proportion being the ATP-binding Cassette (ABC) and Major Facilitator Superfamily (MFS) transporters, which have been implicated in the transport of secondary metabolites including antibiotics [7]. In addition to secondary metabolites, streptomycetes also secret to the environment a mass of proteins through the general secretory (Sec) pathway and the twin-arginine translocation (Tat) pathway [8–10]. These secretory systems are known to facilitate nutrient acquisition. For example, secreted cellulases and chitinases can degrade otherwise insoluble nutrient sources.

Transporters are of critical importance to all living organisms in facilitating metabolism, intercellular communication, biological synthesis and reproduction. They are involved in the uptake of nutrients from the environment, the secretion of metabolites, the efflux of drugs and toxins, the maintenance of ion concentration gradient across membranes, the secretion of macromolecules, such as sugars, lipids, proteins and nucleic acids, signaling molecules, the translocation of membrane proteins, and so on [11]. A Transporter Classification (TC) system has been developed by the Saier group [11, 12]. To date, more than 10,000 non-redundant transport proteins comprising about 750 families are collected in their Transporter Classification Database (TCDB) [13]. These families are divided among seven major classes: Channels/Pores (Class 1), Electrochemical Potential-driven Transporters (Class 2), Primary Active Transporters (Class 3), Group Translocators (Class 4), Transmembrane Electron Carriers (class 5), Accessory Factors Involved in Transport (Class 8), and Incompletely Characterized Transport Systems (Class 9). This classification system has been applied to in-depth studies of transporters in a number of microbial genomes [14–17], and is being adopted in this study for *Streptomyces*.

The availability of genomes from closely related *Streptomyces* species enables comprehensive analysis of the transport protein families in *Streptomyces*. In this study, we report a catalog and comparative genomic analysis of transporters in eleven *Streptomyces* species with complete genome sequences and annotations, including *S. coelicolor* (SCO), *S. avermitilis* (SAV), *S. bingchenggensis* (SBI), *S. cattleya* (SCAT), *S. flavogriseus* (SFLA), *S. griseus* (SGR), *S. hygroscopicus* (SHJG), *S. scabiei* (SCAB), *S. sp. SirexAA-E* (SACTE), *S. venezuelae* (SVEN) and *S. violaceusniger* (STRVI) [7, 18–24]. We identified and classified these *Streptomyces* transporters, using the nomenclature in the TCDB. The class, transmembrane topology and substrate specificity of these transporters are investigated in detail. An improved understanding of *Streptomyces* transporters will bring new insights into the mechanisms underlying the unique and powerful secretion systems of secondary metabolites and proteins in this group of bacteria of enormous economic and biomedical significance.

## Results and discussion

### Abundant transporters are present in eleven *Streptomyces* genomes

Strong material intake and secretion capacity powered by transport systems is an adaptive attribute of soil-dwelling bacteria [1]. We used the coding sequences from eleven *Streptomyces* genomes to query the TCDB [13, 25] using BLASTP and identified 761-1258 transporters in these eleven genomes, which accounted for 10.2 to 13.7 % of each respective proteome (Table 1 and Additional file 1). *S. bingchenggensis*, which has the largest genome, and the largest number of protein-coding genes, has the largest number of transporters, whereas *S. cattleya* contains only 761 transporters, the lowest number and proportion of transporters among the eleven *Streptomyces* species.

**Table 1:** Distribution of transporters in eleven *Streptomyces* genomes

Organisms	Accession ID	Genome size (Mbp)	# ORFs	# Transporters	% Transporters
*S. avermitilis*	NC_003155 (chr)	9.1	7676	989	12.9 %
	NC_004719 (pSAP1)				
*S. bingchenggensis*	NC_016582 (chr)	11.9	10022	1258	12.6 %
*S. cattleya*	NC_016111(chr)	8.1	7475	761	10.2 %
	NC_016113(pSCAT)				
*S. coelicolor*	NC_003888(chr)	9.1	8153	990	12.1 %
	NC_003903 (pSCP1)				
	NC_003904 (pSCP2)				
*S. flavogriseus*	NC_016114 (chr)	7.7	6572	888	13.5 %
	NC_016110 (pSFLA01)				
	NC_016115 (pSFLA02)				
*S. griseus*	NC_010572 (chr)	8.5	7136	975	13.7 %
*S. hygroscopicus*	NC_017765 (chr)	10.4	9108	999	11.0 %
	NC_017766 (pSHJG1)				
	NC_016972 (pSHJG2)				
*S. scabiei*	NC_013929 (chr)	10.1	8746	1021	11.7 %
*S. sp.* SirexAA-E	NC_015953 (chr)	7.4	6357	869	13.7 %
*S. venezuelae*	NC_018750 (chr)	8.2	7453	935	12.5 %
*S. violaceusniger*	NC_015957 (chr)	11.0	8985	989	11.0 %
	NC_015951(pSTRVI01)				
	NC_015952(pSTRVI02)				

### *Streptomyces* transporters show diverse transmembrane topology

**Fig. 1:**
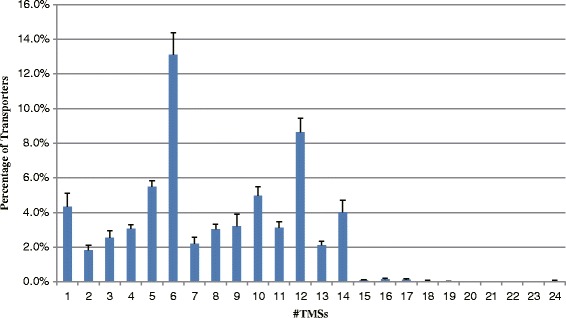
Distribution of transporter topologies in eleven *Streptomyces* genomes. The abbreviations for species are: *S. sp. SirexAA-E* (SACTE), *S. avermitilis* (SAV), *S. bingchenggensis* (SBI), *S. scabiei* (SCAB), *S. cattleya* (SCAT), *S. coelicolor* (SCO), *S. flavogriseus* (SFLA), *S. griseus* (SGR), *S. hygroscopicus* (SHJG), *S. violaceusniger* (STRVI), and *S. venezuelae* (SVEN)

The capacity of a transporter is often associated with the complexity and topology of its transmembrane region(s) where the major events of substrate uptake or output across the cell membranes take place. Using the TMHMM (TransMembrane prediction using Hidden Markov Models) algorithm [26], we performed the transmembrane topology analysis for *Streptomyces* transporters to identify the transmembrane segments (TMSs). The number of TMSs ranges from 0 to 24. The largest number of TMSs observed in a transporter in the eleven *Streptomyces* genomes varies from 16 to 24 (Table 2). Except for intra-/extra-cellular transporters which have no TMS, transporters with 6 and 12 TMSs are predominant. Most transporters with 6 TMSs are ABC transporters (TC 3.A.1), and transporters with 12 TMSs are mainly members of the Major Facilitator Superfamily (MFS) (TC 2.A.1), the Amino Acid-Polyamine-Organocation (APC) superfamily (TC 2.A.3), the Resistance-Nodulation-Cell Division (RND) superfamily (TC 2.A.6) and the ABC superfamily (TC 3.A.1). It is possible that these 12-TMS transporters have arisen from the primordial 6-TMS form via intragenic duplication [27]. Among the transporters with more than 6 TMSs, the transporters with an even number of TMSs are more abundant than those with an odd number of TMSs (Fig. 1). The distribution of TMSs in *S. griseus* transporters is unique: this bacterium has 53 transporters with 9 TMSs, mostly ABC transporters, accounting for 5.4 % of the total transporters. This proportion is significantly higher than that of the other ten sibling species. On the other hand, *S. griseus* has the lowest proportion of 12-TMS transporters (7.3 %), most of which are also ABC transporters. These topology patterns suggest that during the evolution of transporters in *S. griseus*, the “6 + 3” events may be more frequent than the typical “6 + 6” events observed in ten other *Streptomyces* species [27, 28].

**Table 2:** Distribution of topological types of transporters in eleven *Streptomyces* genomes

TMS	SACTE	SAV	SBI	SCAB	SCAT	SCO	SFLA	SGR	SHJG	STRVI	SVEN
0	322	382	482	424	280	344	332	372	392	371	350
1	41	41	55	33	51	47	45	33	28	37	44
2	14	17	18	21	19	19	21	15	16	15	16
3	26	28	32	20	21	26	28	30	22	26	13
4	27	29	31	36	23	32	26	29	36	28	30
5	49	55	62	52	40	58	42	58	56	58	55
6	119	130	201	141	72	135	124	122	116	143	113
7	22	29	23	15	12	24	23	22	20	19	24
8	26	32	35	30	26	34	27	25	35	31	22
9	33	25	36	28	16	35	34	53	30	21	30
10	41	55	62	46	44	54	41	43	48	41	55
11	20	28	42	32	27	30	24	26	40	35	29
12	66	72	109	89	68	89	70	71	97	106	85
13	18	26	24	19	15	20	14	21	25	23	21
14	40	37	43	31	45	38	32	46	35	32	44
15	1	1	1	1	1	1	0	2	0	0	1
16	2	1	1	1	1	1	1	3	2	2	1
17	0	0	1	2	0	2	2	1	1	1	2
18	1	1	0	0	0	0	1	1	0	0	0
19	0	0	0	0	0	0	0	1	0	0	0
24	1	0	0	0	0	1	1	1	0	0	0
Total	869	989	1258	1021	761	990	888	975	999	989	935

Note: SACTE (*S. sp.* SirexAA-E), SAV (*S. avermitilis*), SBI (*S. bingchenggensis*), SCAB (*S. scabiei*), SCAT (*S. cattleya*), SCO (*S. coelicolor*), SFLA (*S. flavogriseus*), SGR (*S. griseus*), SHJG (*S. hygroscopicus*), STRVI (*S. violaceusniger*), SVEN (*S. venezuelae*)

### Transporters in eleven *Streptomyces* genomes can be divided into seven classes and 171 families

**Fig. 2:**
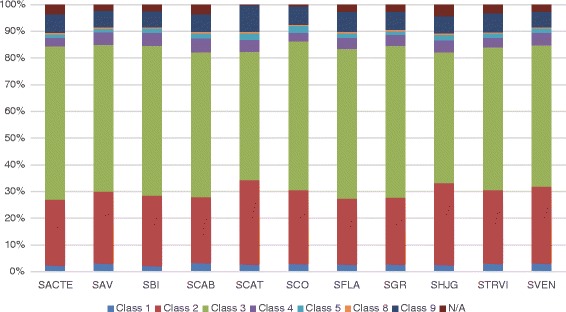
Distribution of transporter types according to the TC system in eleven *Streptomyces* genomes. Class 1: Channels/Proes; Class 2: Electrochemical Potential-driven Transporters; Class 3: Primary Active Transporters; Class 4: Group Translocators; Class 5: Transmembrane Electron Carriers; Class 8: Accessory Factors Involved in Transport; Class 9: Incompletely Characterized Transport Systems; N/A: Not assigned

The *Streptomyces* transporters fall into seven classes and 171 transporter families according to the TCDB system (Table 3 and Additional file 2). The distribution of transporters in each species is depicted in Fig. 2.

**Table 3:** Distribution of *Streptomyces* transporters in each TC class and subclass

Class	Subclass	SACTE	SAV	SBI	SCAB	SCAT	SCO	SFLA	SGR	SHJG	STRVI	SVEN
1: Channels/Proes		22	31	29	34	22	29	26	28	26	31	30
	1.A: α-Type Channels	18	24	20	25	15	22	21	20	21	24	21
	1.B: β-Barrel Porins	3	6	7	8	6	6	4	5	4	6	6
	1.C: Pore-Forming Toxins (Proteins and Peptides)	1	1	1	1	1	1	1	3	1	1	3
	1.I: Membrane-bounded channels	0	0	1	0	0	0	0	0	0	0	0
2: Electrochemical Potential-driven Transporters		212	266	330	251	239	274	217	242	305	271	269
	2.A: Porters (uniporters, symporters, antiporters)	212	266	328	251	239	274	217	242	305	271	269
	2.C: Ion-gradient-driven energizers	0	0	2	0	0	0	0	0	0	0	0
3: Primary Active Transporters		500	544	705	553	365	552	498	555	489	528	494
	3.A: P-P-bond-hydrolysis-driven transporters	455	492	656	505	304	497	451	508	433	476	449
	3.B: Decarboxylation-driven transporters	6	6	5	6	10	7	6	6	6	4	6
	3.D: Oxidoreduction-driven transporters	39	46	43	42	51	48	41	41	50	47	39
	3.E: Light absorption-driven transporters	0	0	1	0	0	0	0	0	0	1	0
4: Group Translocators		27	46	62	54	35	30	36	40	46	37	43
	4.A: Phosphotransfer-driven group translocators	5	7	4	5	2	8	8	6	5	7	6
	4.B: Nicotinamide ribonucleoside uptake transporters	1	1	0	1	1	1	3	3	1	0	3
	4.C: Acyl CoA ligase-coupled transporters	21	38	58	48	32	21	25	31	40	30	34
5: Transmembrane Electron Carriers		12	13	21	19	18	26	15	13	20	16	16
	5.A: Transmembrane 2-electron transfer carriers	12	12	21	18	17	26	14	13	19	15	16
	5.B: Transmembrane 1-electron transfer carriers	0	1	0	1	1	0	1	0	1	1	0
8: Accessory Factors Involved in Transport		4	4	5	6	5	5	5	6	6	4	4
	8.A: Auxiliary transport proteins	4	4	5	6	5	5	5	6	6	4	4
9: Incompletely Characterized Transport Systems		60	63	74	67	75	67	68	66	63	69	55
	9.A: Recognized transporters of unknown biochemical mechanism	27	25	44	27	33	31	32	35	27	33	25
	9.B: Putative transport proteins	33	38	30	40	42	36	35	31	36	36	30
	9.C: Functionally characterized transporters lacking identified sequences	0	0	0	0	0	0	1	0	0	0	0
N/A		32	22	32	37	2	7	23	25	44	33	24
Total		869	989	1258	1021	761	990	888	975	999	989	935

The Primary Active Transporters (Class 3) is the most abundant class of transporters in *Streptomyces*, which includes 365-705 transporters (representing about 48.0-57.5 % of the total transport machinery). This class of transporters plays important roles in various aspects of bacterial life cycle, especially in the import and export of secondary metabolites, and cation transportation.

Class 2 transporters, the electrochemical potential-driven transporters, are also widely found in *Streptomyces*. 212-330 transporters in eleven *Streptomyces* genomes belong to this class, which account for 24.4 %-31.4 % of all the transporters. The porters in this class include uniporters, symporters and antiporters. The most abundant family, MFS, in Class 2 transporters has been implicated in drug efflux. Lineage-specificity is also observed in this class of transporters. For example, *S. bingchenggensis* possesses two Ion-gradient-driven Energizers (TC 2.C), while the other ten *Streptomyces* species only have Porters (uniporters, symporters, antiporters) (TC 2.A).

Class 1 transporters are not abundant, but are functionally important for *Streptomyces*. 22-34 channel/pore transporters are present in these eleven genomes, accounting for 2.3 %-3.2 % of all the transporters. The majority of these channel-type proteins are alpha-type channels (TC 1.A), which have been implicated in stress responses of Gram-positive bacteria, especially responses to osmotic pressure [27]. A small number of proteins belong to β-type porins and a fewer are putative Channel-Forming Toxins (TC 1.C). The membrane-bounded channel (TC 1.I) subclass is rare in *Streptomyces*; only *S. bingchenggensis* has a transport protein from this subclass.

Classes 4, 5, and 8 are relatively less abundant. About 3.0 %-5.3 % of all the transport proteins are Class 4 transporters. Two major subclasses observed in Class 4 are the PTS Glucose-Glucoside (Glc) family (4.A.1) and the Fatty Acid Transporter (FAT) family (4.C.1), which are responsible for the transport of glucoses-glucosides and fatty acids, respectively. Notably, *S. cattleya*, which has the smallest repertoire of transporters among the eleven *Streptomyces*, does not seem to contain any Glc transporters; it remains unknown if it uses an alternative system. Only 12-21 members of the Class 4 transporters, the Transmembrane Electron Carriers, are found in *Streptomyces*. Two subclasses are present, including the Prokaryotic Molybdopterin-containing Oxidoreductase (PMO) family (TC 5.A.3) and the Prokaryotic Succinate Dehydrogenase (SDH) family (TC 5.A.4), which transfer electrons mainly by redox reactions. Class 8, the Accessory Factors Involved in Transport, is the least abundant transporter class (0.4 %-0.7 %) in *Streptomyces*.

A significant number (60-75) of transporters in *Streptomyces* can be grouped into Class 9, an incompletely characterized class. While their exact physiological roles are yet to be elucidated, they might be involved in the transport of ions, implicated by their sequence similarities with the members of the HlyC/CorC (HCC) family (TC 9.A.40), and the Tripartite Zn^2+^ Transporter (TZT) family (TC 9.B.10).

### Examples of important transporter families

Many of the 171 transporter families are involved in the transfer of ions, saccharides, amino acids, polypeptides, proteins, drugs, toxins and other compounds. The two most abundant and perhaps also the most important families are in the ABC (TC 3.A.1) and MFS (TC 2.A.1) superfamilies. They are responsible for the secretion of a wide array of antibiotics in *Streptomyces* [29, 30].

#### The ABC transporters

32.7 %-47.5 % (249-597) of all the transport proteins in the eleven *Streptomyces* genomes are members of ABC superfamily. ABC transporters are characterized by a conserved ATP hydrolyzing domain for energy provision, pore-forming membrane-integrated domain(s), and a substrate-binding domain [31, 32]. The ABC transport system is composed of the intake system and the efflux system.

The 30 intake families (TC 3.A.1-3.A.33) that we identified in the *Streptomyces* genomes are specialized in the uptake of diverse nutrient substances. This intake system includes families of Carbohydrate Uptake Transporters (TC 3.A.1.1, 3.A.1.2) that transport saccharides, Polar Amino Acid Uptake Transporters and Hydrophobic Amino Acid Uptake Transporters (TC 3.A.1.3, 3.A.1.4) that transfer amino acids, Polyamine/Opine/ Phosphonate Uptake Transporters and Quaternary Amine Uptake Transporters (TC 3.A.1.11, 3.A.1.12) that transfer amine substances, Iron Chelate Uptake Transporters and Manganese/Zinc/Iron Chelate Uptake Transporters (TC 3.A.1.14, 3.A.1.15) that transfer metal ions.

Unlike the intake system, the 35 *Streptomyces* efflux families are involved in the transport of macromolecular substances. These transporters are believed to be essential for *Streptomyces* due to their roles in drug efflux and protein secretion. The drug efflux system regulates various aspects of the response to drug compounds mediated by Drug Exporters (TC 3.A.1.105, 3.A.1.117, 3.A.1.119, 3.A.1.135), Drug Resistance ATPases (TC 3.A.1.120, 3.A.1.121), Macrolide Exporters (TC 3.A.1.122), β-Exotoxin I Exporters (TC 3.A.1.126), Multidrug Resistance Exporters (TC 3.A.201) and Pleiotropic Drug Resistance transporters (TC 3.A.1.205). Potent protein transport in *Streptomyces* is regulated by Protein/Peptide Exporters (TC 3.A.1.109, 110, 111, 112, 123, 124, 134), Lipoprotein Translocases (TC 3.A.1.125), AmfS Peptide Exporters (TC 3.A.1.127), and SkfA Peptide Exporters (TC 3.A.1.128).

#### The MFS transporters

Unlike the ABC transporters, the MFS transporters are driven by an electrochemical potential formed by ion concentration gradients across the cytomembrane [30]. There are 90-169 (10.1 %- 15.0 %) MFS transporters in eleven *Streptomyces* genomes. *Streptomyces* possesses 39 subfamilies of MFS transporters, including 20 intake systems, 13 efflux systems and 6 systems whose transport direction is unknown. The substances transported by the intake systems are mainly saccharides and organic acids.

One of the most important roles of the MFS transporters is drug efflux [30]. Diverse subfamilies of drug efflux MFS transporters are present in *Streptomyces*, with varying mechanisms of action, including Drug:H^+^ Antiporters (TC 2.A.1.2, 2.A.1.3, 2.A.1.21), Aromatic Compound/Drug Exporters (TC 2.A.1.32), Fosmidomycin Resistance transporters (TC 2.A.1.35), Acriflavin-sensitivity transporters (TC 2.A.1.36), and Microcin C51 Immunity Proteins (TC 2.A.1.61), to name a few.

### The wide distribution of substrates for *Streptomyces* transporters

**Fig. 3:**
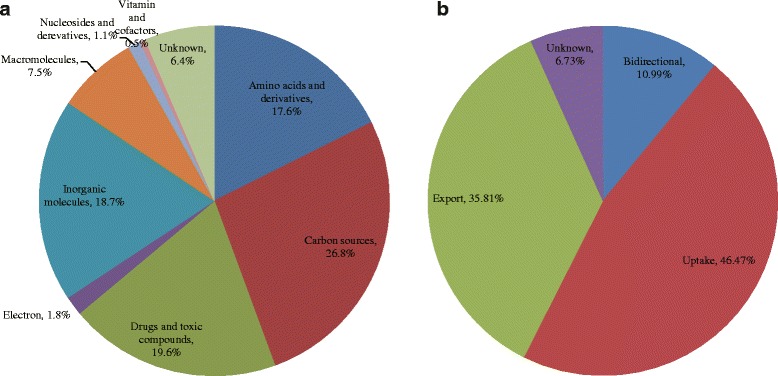
**a** Distribution of substrate types and (**b**) predicted polar characteristics: bidirectional transport, uptake or export in eleven *Streptomyces* genomes

The capacity of the complex and powerful transporter system in *Streptomyces* is evidenced by the broad scope of the substrates being transported. Figure 3a shows the distribution of transporters that transport different type of substrates in *Streptomyces*, including carbon sources, drugs, toxicants, electrons, inorganic molecules, macromolecules, amino acids and derivatives, nucleotides and derivatives, vitamins, and accessory factors. The carbon source transporters are the most abundant, with their proportion of all the transport proteins ranging from 21.7 to 31.6 % in eleven genomes. Notably, the substrates of an average of 6.4 % of the transporters in *Streptomyces* genomes examined cannot be determined based on genomic analysis, and await advanced structural and biochemical characterization.

*Streptomyces* transporters can be divided into three classes, uptake, efflux and bidirectional, according to the direction of the substrates transported (Fig. 3b). Among the transporters of the eleven *Streptomyces* genomes, on average 46.5 % are involved in the uptake of substrates, 35.8 % are involved in the efflux of substrates, and 11.0 % are in charge of the bidirectional transport of substrates. The direction of 6.7 % of these proteins remains undetermined.

### *Streptomyces* have lineage-specific protein secretion systems

**Fig. 4:**
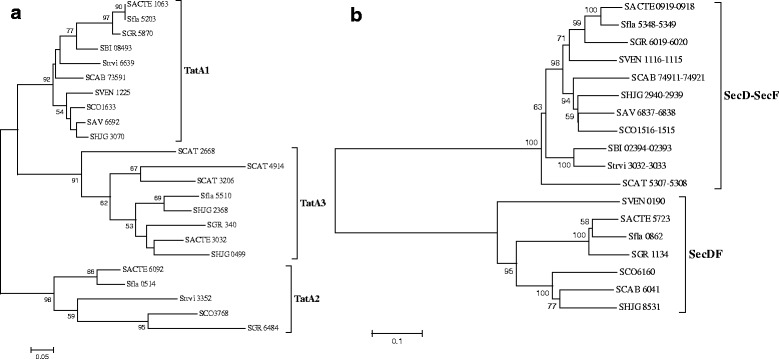
**a** Phylogenetic tree of the TatA system. **b** Phylogenetic tree of the SecD/SecF (**b**) system in eleven *Streptomyces* genomes. The trees were constructed using the neighbor-joining method by MEGA6 [43]. The Maximum Parsimony and Maximum Likelihood methods gave virtually the same topology (data not shown)

*Streptomyces* have two major lineage-specific protein transport systems, the Tat system (TC 2.A.64) and the Sec system (TC 3.A.5) [8, 9]. The Tat system was shown to be related to the pathogenicity of pathogenic bacteria [33]. In *S. scabies*, the transporters in the Tat pathway secrete several toxicity-associated proteins [34]. While the key component proteins of the Tat system, TatA, TatB and TatC, are present in all eleven *Streptomyces* genomes we looked at, lineage-specificity is clearly shown with respect to the copy number variation of these genes (Table 4). Only one copy of the *tatB* and *tatC* genes is present in nine *Streptomyces* genomes; *S. flavogriseus* has two copies of the *tatB* genes and *S. hygroscopicus* has two copies of the *tatC* genes. The copy number of the *tatA* gene ranges from one to three in eleven genomes (Table 4). Phylogenetic analysis shows that the multiple copies of the *tatA* genes may have different evolutionary origins and can be divided into three independent clades, namely *tatA1*, *tatA2* and *tatA3* (Fig. 4a). The *tatA* paralogous genes in the majority of the *Streptomyces* genomes belong to different clades. Notably, all the three *tatA* paralogous genes in *S. cattleya* are clustered into the *tatA3* clade, indicative of recent gene duplication events.

**Table 4:** The Tat translocation system in *Streptomyces* (TC 2.A.64)

Species	*tatA1*	*tatA2*	*tatA3*	*tatB1*	*tatB2*	*tatC1*	*tatC2*
SACTE	SACTE_1063	SACTE_6092	SACTE_3032	SACTE_4381		SACTE_1062	
SAV	SAV_6692			SAV_3114		SAV_6693	
SBI	SBI_08493			SBI_04079		SBI_08494	
SCAB	SCAB_73591			SCAB_31121		SCAB_73601	
SCAT	SCAT_3206	SCAT_2668	SCAT_4914	SCAT_4007		SCAT_5184	
SCO	SCO1633	SCO3768		SCO5150		SCO1632	
SFLA	Sfla_5203	Sfla_0514	Sfla_5510	Sfla_5507	Sfla_2146	Sfla_5204	
SGR	SGR_5870	SGR_6484	SGR_340	SGR_2375		SGR_5871	
SHJG	SHJG_2368	SHJG_3070	SHJG_0499	SHJG_6250		SHJG_2367	SHJG_3069
STRVI	Strvi_6639	Strvi_3352		Strvi_1468		Strvi_6638	
SVEN	SVEN_1225			SVEN_4796		SVEN_1224

Similarly, the Sec system is also species-specific. This system includes SecA, SecY, SecE, SecG, SecD, SecF, YajC, FtsY, etc. [35], all of which are highly conserved in *Streptomyces* (Table 5). There is only one copy of the *secE*, *secG*, *secD*, *secF*, *yajC* and *ftsY* genes in each of the eleven *Streptomyces* genomes. Interestingly, there is a second set of *secA2*/*secY2* genes in several species, which may be involved in the secretion of proteins with specific functions, for example, the secretion of toxic proteins [36]. In *S. avermitilis*, for instance, there are two copies of the *secA* genes, and *S. venezuelae* has two copies of the *secY* genes.

**Table 5:** The Sec translocation system in *Streptomyces* (TC 3.A.5)

Species	*secA1*	*secA2*	*secY*	*secY2*	*secE*	*secG*
SACTE	SACTE_2472		SACTE_3988		SACTE_3949	SACTE_1366
SAV	SAV_5071	SAV_2565	SAV_4312		SAV_4908	SAV_6299
SBI	SBI_06502		SBI_06209		SBI_06158	SBI_08032
SCAB	SCAB_55371		SCAB_36741		SCAB_37261	SCAB_69731
SCAT	SCAT_2009		SCAT_3612		SCAT_3559	SCAT_1102
SCO	SCO3005		SCO4722		SCO4646	SCO1944
SFLA	Sfla_3902		Sfla_2503		Sfla_2541	Sfla_4882
SGR	SGR_4531		SGR_2814		SGR_2876	SGR_5576
SHJG	SHJG_4468		SHJG_5817		SHJG_5775	SHJG_3400
STRVI	Strvi_8396		Strvi_0893		Strvi_0854	Strvi_7031
SVEN	SVEN_2748		SVEN_4399	SVEN_0354	SVEN_4338	SVEN_1573
Species	*secD*	*secF*		*secDF*	*yajC*	*ftsY*
SACTE	SACTE_0919	SACTE_0918		SACTE_5723	SACTE_0920	SACTE_4801
SAV	SAV_6837	SAV_6838			SAV_6836	SAV_2654
SBI	SBI_02394	SBI_02393			SBI_02395	SBI_03477
SCAB	SCAB_74911	SCAB_74921		SCAB_6041	SCAB_74901	SCAB_26291
SCAT	SCAT_5307	SCAT_5308			SCAT_5306	SCAT_4417
SCO	SCO1516	SCO1515		SCO6160	SCO1517	SCO5580
SFLA	Sfla_5348	Sfla_5349		Sfla_0862	Sfla_5347	Sfla_1718
SGR	SGR_6019	SGR_6020		SGR_1134	SGR_6018	SGR_1898
SHJG	SHJG_2940	SHJG_2939		SHJG_8531	SHJG_2941	SHJG_6701
STRVI	Strvi_3032	Strvi_3033			Strvi_3031	Strvi_1937
SVEN	SVEN_1116	SVEN_1115		SVEN_0190	SVEN_1117	SVEN_5276

The evolutionary pattern in the *secD* and the *secF* genes is particularly interesting (Fig. 4b). In bacteria, these genes encode accessory factors in the Sec pathway that can accelerate the translocation of protein substrates. There are two forms of the *secD* and *secF* genes: in the first form, these two genes are adjacent but separate, while in the second form, the two genes are fused into a single *secDF* gene. The fused *secDF* is present in seven *Streptomyces* genomes. Unlike most bacteria that have one of the two forms, the majority of *Streptomyces* species have both the separated form and the fused form [37]. The acquisition of a second copy may confer a selective advantage to *Streptomyces* by enhancing the capacity and the effectiveness of protein transport.

## Conclusions

Comparative genomic analyses of eleven *Streptomyces* genomes revealed an abundant repertoire of 761-1258 transporters, belonging to seven transporter classes and 171 transporter families. The powerful transport systems in *Streptomyces* play critical roles in drug efflux, protein secretion and stress response. A better understanding of transport systems will allow enhanced optimization of production processes for both pharmaceutical and industrial applications of *Streptomyces*.

## Methods

### Data

The completed whole genome data of the eleven *Streptomyces* species (Table 1), including amino acid sequences and functional annotations of all the proteins were downloaded from the NCBI database (http://www.ncbi.nlm.nih.gov/genome/browse/). The transporter classification and amino acid sequences of all classified transporters were downloaded from the TCDB database (http://www.tcdb.org/) [13]. We also collected data from the TransporterDB database [38] (http://www.membranetransport.org/) which included the transporter classification data of *S. coelicolor* and *S. avermitilis*, and from the Transporter Inference Parser database [39] (http://biocyc.org/), which identified transporter according to their function annotation and included the relevant data of *S. coelicolor*, *S. avermitilis*, *S. griseus* and *S. scabies*.

### Identification and classification of transporters

The BLASTP search of all the proteins in eleven *Streptomyces* species versus all the transport proteins in TCDB database was conducted to identify transporters in *Streptomyces* that are homologs to known and predicted transporters in the TCDB [13, 25]. The threshold for homologous genes was set as follow: E-value ≤ 10^-5^, similarity ≥ 50 %, and the sequence coverage ≥ 30 %. We classified a *Streptomyces* transporter based on its homologous gene with known function in the TCDB that had the lowest expected value, the highest similarity score and the highest coverage. The classification of *Streptomyces* transporters in the TransporterDB and the Transporter Inference Parser, the annotations and the conserved domain information helped to filter false negative and false positive predictions. The Pfam search program based on the Hidden Markov Models (HMMs) (http://pfam.xfam.org/) [40] was used to identify conserved structure domains of *Streptomyces* transporters, with Pfam GA as the threshold. TMHMM (http://www.cbs.dtu.dk/services/TMHMM/) [26] was used to analyze the transmembrane structures and the number of putative TMSs of *Streptomyces* transporters.

On the basis of the degree of similarities with known or predicted transporters in the TCDB, as well as the conserved domains and the number and location of TMSs, we further classified the *Streptomyces* transporters into families and subfamilies of homologous transporters according to the TC system [13]. The TC number generally has five components: V.W.X.Y.Z, representing the transporter class, subclass, family, subfamily and the substrate or range of substrates transported [11, 12]. Most *Streptomyces* transporters were classified at the transporter family level. The transporters in superfamilies such as ABC and MFS were classified at the subfamily level.

The substrate and transport direction of each *Streptomyces* transporter was predicted based on homology to functionally characterized transporters in the TCDB. Classification of a putative transporter into a family or subfamily according to the TC system allows for the prediction of substrate types and transport direction with confidence [13, 17, 41].

### Phylogenetic analysis of transport protein families

Multiple sequence alignments were obtained using Clustal X 2.1 [42]. Phylogenetic trees were reconstructed using MEGA6 with neighbor-joining (NJ), maximum parsimony (MP) and maximum likelihood (ML) methods [43].

## Additional files

Additional file 1: A detailed description of transporters in eleven *Streptomyces* genomes. The file includs protein IDs, names, annotations, protein lengths, Pfam domains, number of TMSs, and their homologs in TCDB with the BLASTP E-value. (XLSX 918 kb) Additional file 2: The classification of *Streptomyces* transporters. (XLSX 76 kb)
